# Trends in site of pediatric firearm death in the United States

**DOI:** 10.1093/aje/kwag192

**Published:** 2026-08-13

**Authors:** Angelin Tresa Mathew, Urvish Jain, Bhav Jain, Edward Christopher Dee, Myron Rolle, Megan Ranney

**Affiliations:** Department of Molecular, Cellular, and Developmental Biology, Yale University, New Haven, CT, United States; Bennett School of Public Policy, University of Cambridge, Cambridge, United Kingdom; Perelman School of Medicine, University of Pennsylvania, Philadelphia, PA, United States; Department of Health Policy, Stanford University School of Medicine, Stanford, CA, United States; Department of Radiation Oncology, Memorial Sloan Kettering Cancer Center, New York, NY, United States; Department of Pediatric Neurosurgery, Johns Hopkins Medical Center, Baltimore, MD, United States; Yale School of Public Health, Yale University, New Haven, CT, United States

**Keywords:** firearms, site of death, epidemiology

## Introduction

Firearms are the leading cause of death in children in the United States, and the United States accounts for the majority of children’s firearm deaths among high-income countries.^1^ Firearm deaths occur in diverse locations, primarily including inpatient, outpatient, on scene, and on or before arrival to medical care. Others have examined patterns of location of firearm death among adults in the United States as a proxy for lethality, injury severity, prehospital response effectiveness, and access to emergency medical care.^1-5^ To our knowledge, trends in the site of firearm death based on sociodemographic factors among children have not been examined. Understanding where children die from firearm injuries is clinically meaningful because the site of death may reflect injury lethality, prehospital response, and access to timely emergency care, all of which may vary by race, ethnicity, and sex.

## Methods

Multivariable logistic regression using the Centers for Disease Control and Prevention (CDC) dataset, CDC WONDER, was performed for pediatric (ages 1-19 years) firearm deaths from assaults, unintentional injuries, and unknown intent from 2003-2021. Independent variables include age, race, Hispanic ethnicity, sex, and year. Our primary outcome for this study was location of death as defined by the CDC, including inpatient, outpatient, dead on arrival, home, hospital, nursing, other, and unknown. Home and other were classified as death at the scene, based on prior literature.^2^ Linear regression was performed on each location over time and deaths at scene by each age group over time. *International Classification of Diseases, Tenth Revision*, codes X85-Y09 were used. Self-inflicted deaths were not considered, because they almost exclusively occur at the scene. This study was exempt from IRB review given the use of publicly available, de-identified data and conducted in accordance with EQUATOR guidelines. STATA SE 17.0 was used for all analysis.

## Results

We excluded 233 observations (0.51%) due to missing data. A total of 44,658 children died by firearm injury in the United States between 2003 and 2021. The percentage of deaths on scene and at outpatient or emergency department (ED) settings increased over time (*P* < .01). The percentage of deaths on arrival decreased over time (*P* < .001) (Figure 1A).

**Figure 1:**
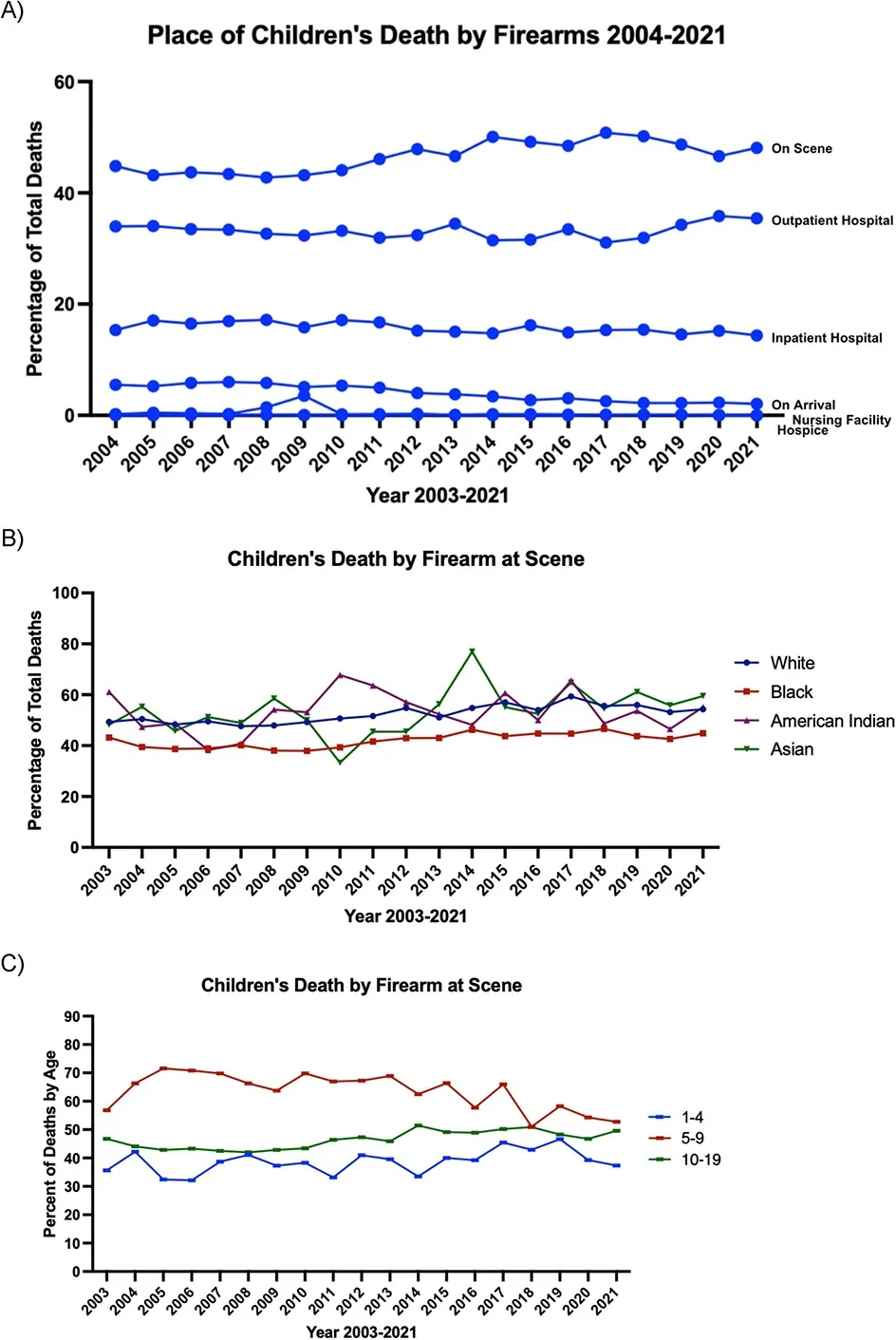
(A) Overall trends in site of death over time for children from firearm injuries (B) Racial disparities in at-scene deaths over time. (C) Children’s deaths by age at scene.

Male children were significantly less likely to be dead on the scene (Table 1) (adjusted odds ratio [aOR] = 0.63 [95% CI, 0.59, 0.66]; *P* < .001); boys were more likely to die as hospital inpatients (aOR = 1.22 [95% CI, 1.15, 1.32]; *P* < .001), outpatients (aOR = 1.46 [95% CI, 1.37, 1.53]; *P* < 0.001) or be dead upon arrival (aOR = 1.41 [95% CI, 1.22, 1.63]; *P* < .001). Non-White children—including Black (aOR = 1.74 [95% CI, 1.65, 1.84]; *P* < .001), Asian (aOR: 1.25 [95% CI, 1.06, 1.47]; *P* < .001), and Hispanic (aOR: 1.69 [95% CI, 1.59, 1.80]; *P* < .001) children—were more likely to die in outpatient or ED settings than White children. Black children were least likely to be dead on the scene (aOR = 0.52 [95% CI, 0.49–0.55]; *P* < .001) but more than twice as likely to be dead upon arrival (aOR = 2.23 [95% CI, 2.01–2.70]; *P* < .001) (Figure 1B). The percentage of children aged 1-4 years who died on scene increased over time (*P* < .01), whereas the percentage of children aged 5-9 years who died on scene decreased (*P* < 0.05) (Figure 1C).

**Table 1:** Association between location of death by firearm in pediatric patients and decedent characteristics, 2003-2021.

**Characteristic**	**Inpatient vs all other locations of death** ^**a**^	**Outpatient and emergency department vs all other locations of death** ^**a**^	**Dead on arrival vs all other locations of death** ^**a**^	**Scene vs all other locations of death** ^**a**^
No. of patients	7094	14 946	1852	20 887
Age, y				
≤ 4	1 (Reference)	1 (Reference)	1 (Reference)	1 (Reference)
5-9	0.40 (0.34, 0.46)^***^	0.60 (0.53, 0.68)^***^	0.74 (0.54, 0.99)^*^	2.72 (2.43, 3.04)^***^
10-19	0.51 (0.47, 0.56)^***^	0.85 (0.79, 0.91)^***^	0.81 (0.67, 0.94)^**^	1.85 (1.72, 1.98)^***^
Sex				
Female	1 (Reference)	1 (Reference)	1 (Reference)	1 (Reference)
Male	1.22 (1.14, 1.31)^***^	1.46 (1.38, 1.54)^***^	1.41 (1.22, 1.63)^***^	0.63 (0.59, 0.66)^***^
Race				
White	1 (Reference)	1 (Reference)	1 (Reference)	1 (Reference)
Black	1.12 (1.05, 1.20)^***^	1.74 (1.65, 1.84)^***^	2.33 (2.01, 2.70)^***^	0.52 (0.49, 0.55)^***^
American Indian	1.01 (0.80, 1.27) n.s.	1.17 (0.97, 1.41) n.s.	1.58 (1.01, 2.46)^*^	0.85 (0.72, 1.01) n.s.
Asian	1.14 (0.94, 1.39) n.s.	1.25 (1.06, 1.47)^**^	0.60 (0.34, 1.08) n.s.	0.83 (0.72, 0.96)^*^
Ethnicity				
Non-Hispanic	1 (Reference)	1 (Reference)	1 (Reference)	1 (Reference)
Hispanic	1.14 (1.05, 1.23)^***^	1.68 (1.59, 1.80)^***^	1.03 (0.87, 1.23) n.s.	0.60 (0.57, 0.64)^***^
Time (year)	0.99 (0.99, 1.00)^***^	1.00 (1.00, 1.01) n.s.	0.93 (0.92, 0.94)^***^	1.02 (1.01, 1.02)^***^

^***^ *P* < 0.001, ^**^P < 0.01, ^*^P < 0.05, n.s., not significant.

^a^Data are shown as adjusted odds ratio over time with 95% CI.

## Discussion

Children’s rate and frequency of death by firearm have significantly increased in the United States over the past 2 decades. Similar to adults, the location of death has shifted, with an increasing proportion of deaths occurring outside of a trauma center.^2^ Older children aged 5-19 years were more likely to die on scene, potentially due to sustaining more fatal firearm injuries.^2^ This is especially notable given that younger children have lower functional reserve, blood volume, and innate ability to decompensate quicker.^6^

Female children are more frequently victims of firearm injuries from family violence, which may explain their higher frequency of deaths at the scene.^3^ Children from racial and ethnic minority backgrounds had an increased likelihood of dying while receiving medical care for firearm injuries, which could stem from a number of factors, including differences in the types of injuries sustained or in baseline rates of firearm encounters.^7-9^ Others have reported that hospitals serving predominantly minority communities often lack trauma centers and advanced emergency response resources; these disparities may also contribute to differences in the location of fatalities.^3^^,^^4^

Due to the retrospective nature of the data, we are unable to draw causal conclusions. Furthermore, first responders may have variable protocols by state on classification of death location. Future work should examine correlations among injury severity, case fatality, and demographics; between trends in location of death and proximity of the shooting to a trauma center; and between lethality and firearm or magazine type. Concerted efforts from civilians, clinicians, researchers, and policymakers are necessary to address this health crisis affecting American children.^5^

## Conflicts of interest

None declared.

## Data Availability

All data are publicly available from the CDC and National Bureau of Economic Research at https://www.nber.org/research/data/vital-statistics-mortality-data-nber
